# Retraction: Interferon-β Induces Cellular Senescence in Cutaneous Human Papilloma Virus-Transformed Human Keratinocytes by Affecting p53 Transactivating Activity

**DOI:** 10.1371/journal.pone.0214341

**Published:** 2019-03-15

**Authors:** The PLOS ONE Editors

After publication of this [1] article, the following concerns were raised regarding the figures:

- Fig 3, p53 and p21 panels contain vertical discontinuities after lane 9 and l.c. panel contains vertical discontinuities after lane 7.
- Fig 4A contains a horizontal discontinuity between PML and p53 panels.
- Fig 4B, lane 3 within the l.c. panel contains an area covered by a white box (lower right) and appears to have a horizontal discontinuity or splice line above the band.
- Fig 6A p53 panel contains vertical discontinuities after lane 8.
- Fig 6C lys320 panel contains vertical discontinuities after lane 13, lys373/382 panel contains vertical discontinuities after lane13, l.c panel contains vertical discontinuities after lane 12.
- Fig 6D, totp53, ser6, ser392, lys320, and ΔNp73 panels contain vertical discontinuities after lane 2. The totp53 panel also appears to have vertical discontinuities after lane 1.
- Fig 6D, similarities are noted between lanes 1 and 2 of the totp53 panel.
- Fig 6E l.c. panel: lane 1 appears similar to lane 9; lanes 2, 3 appear similar to lanes 11, 12; lane 4 appears similar to lane 10; and lanes 5, 6 appear similar to lanes 7, 8.

For several figures in the article, the brightness and contrast do not allow evaluation of the background for the Western blot data.

The authors provided underlying raw blots for some of the figures; these data clarified the concerns about Figs 3, 4B and 6C. For the Fig 3 p53, p21 and l.c. panels, the authors explained that protein samples were loaded across two gels and composite images were generated. For Fig 4B l.c., parts of the background in lane 3 of the l.c. panel were masked to cover non-specific markings. For the Fig 6C lys 373/382 and lys 320 panels, the authors explained that the K16 and LXSN control proteins were loaded on the same gel but in a different order to that described in the article and a composite image was generated for the purposes of figure presentation.

The authors are unable to provide the raw unadjusted blots underlying Fig 6A l.c., Fig 6D p53, lys 320, and ΔNp73, Fig 6E ΔNp73 and l.c. panels. For Fig 6C l.c. panel lanes 7–14, the raw blot provided is from separate studies using the same cellular extracts. For Fig 6D p53 and 6D lys 320 panels the authors provided raw blots underlying replicate experiments.

The institution investigated these concerns and concluded that these do not affect the reliability of the data or the conclusions of the article. However, in light of the absence of underlying data to fully resolve all image concerns, the integrity and reliability of the published data remains in question, and the *PLOS ONE* Editors retract this article.

MC, SV, RA, MT, ZP, GV, EA, GF, GR did not agree with retraction.
